# Missing a Lipiodol-laden hepatocellular carcinoma?: Silent iatrogenic hepatoduodenal fistula after transarterial chemoembolization: a case report

**DOI:** 10.1097/MD.0000000000039632

**Published:** 2024-09-13

**Authors:** Byung Chan Lee, Hyoung Ook Kim, Chan Park, Inwoo Choi, Seung Wan Kang

**Affiliations:** a Department of Radiology, Chonnam National University Hwasun Hospital and Chonnam National University Medical School, Hwasun-eup, Hwasun-gun, Jeollanam-do, South Korea; b Department of Radiology, Chonnam National University Hospital and Chonnam National University Medical School, Gwangju, South Korea.

**Keywords:** hepatocellular carcinoma, hepatoenteric fistula, transarterial chemoembolization

## Abstract

**Rationale:**

Owing to the abundant collateral blood supply to the duodenum, the development of a hepatoduodenal fistula after transarterial chemoembolization (TACE) is an extremely rare complication that usually requires hospitalization and intensive medical intervention. Here, we report a case of a silent hepatoduodenal fistula following TACE.

**Patient concerns:**

A 74-year-old man with a history of alcoholic liver cirrhosis and type 2 diabetes. He had undergone a partial hepatectomy due to hepatocellular carcinoma (HCC) 7 years ago. In addition, he had undergone 4 TACEs for the treatment of recurrent HCCs but still had a viable tumor in S4b of the liver, which abuts the duodenal 1st portion.

**Diagnoses:**

HCC.

**Interventions:**

The patient underwent a 5th TACE and was discharged from the hospital without major adverse events.

**Outcomes:**

Follow-up computed tomography scans showed a 2 cm–sized air cavity instead of a compact Lipiodol-laden tumor in S4b, which had shrunk over time. The patient had experienced a fluctuating nonspecific mild fever for 3 months, with improvements in symptoms and laboratory findings following conservative treatment alone.

**Lessons:**

Hepatic fistulas may arise following TACE for HCCs near the gastrointestinal tract and may be present with nonspecific symptoms. This case suggests that increased efforts should be directed toward achieving selective embolization when treating HCC adjacent to the gastrointestinal tract, with close monitoring required after treatment.

## 1. Introduction

Transarterial chemoembolization (TACE) is the primary treatment for patients diagnosed with intermediate-stage hepatocellular carcinoma (HCC). It utilizes a dual mechanism of targeted chemotherapy delivery and ischemic insult to the tumor vasculature.^[1]^ Although TACE is widely used due to its therapeutic efficacy, there have been some reports of rare complications, such as duodenal perforations or hepatoduodenal fistulas.^[2–5]^ The location of the HCC can influence the extrahepatic arterial supply, particularly if it is situated in the liver’s bare area, an exophytic tumor, or present with extrahepatic tumor invasion. Furthermore, the probability of an extrahepatic artery providing blood supply to the HCC increases in cases where the patient has undergone multiple TACE procedures or has a history of surgery.^[6]^

This case report presents a unique instance of a silent hepatoduodenal fistula developing after TACE in a patient with HCC—supplied by extrahepatic arteries—without the usual precursors of excessive tumor size or abscess formation.

## 2. Case report

**Figure 1.**
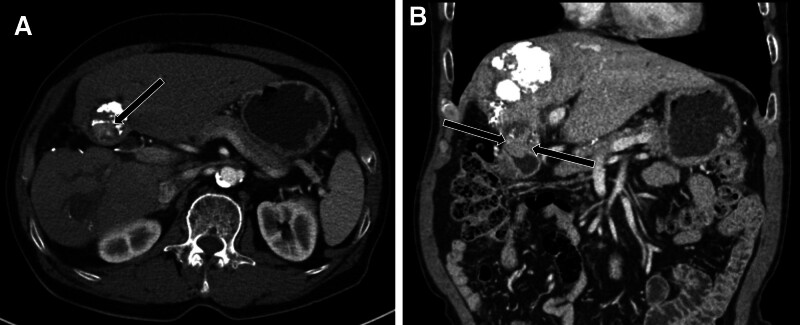
Preprocedural CT images obtained before the 5th TACE. (A) Axial plane arterial phase images of a viable tumor in S4b of the liver (arrow). (B) Coronal reformatted images of a diminished fat plane between the tumor and the duodenal 1st portion (arrows) with suspected tumor invasion. CT = computed tomography, TACE = transarterial chemoembolization.

**Figure 2.**
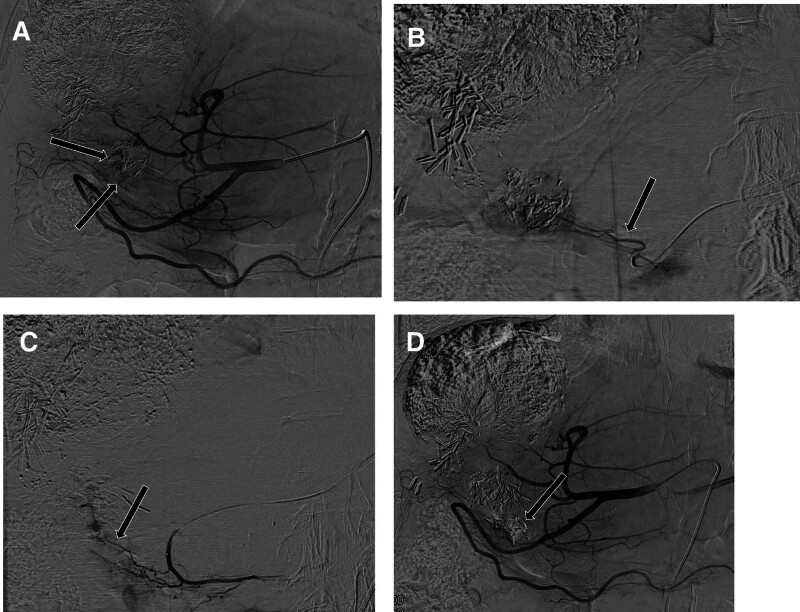
(A) Digital subtraction angiogram of the common hepatic artery with tumor staining (arrows) and supplied by the small branches of the gastroduodenal artery. (B and C) Superselective angiogram of tumor feeders from the gastroduodenal artery (arrows). (D) Completion angiogram of a compact lipiodolized tumor without a viable portion. Extravasation of the chemoembolic agent due to vessel rupture during procedure was noted (arrow).

A 74-year-old man was admitted to our hospital for a 5th session of TACE. He had a history of alcoholic liver cirrhosis and type 2 diabetes and had undergone a partial hepatectomy 7 years ago because of HCC. Initially, he had a 7.8 cm–sized HCC in the right hepatic lobe and a 4 cm–sized HCC in S4b of the liver. He subsequently received 2 rounds of drug-eluting bead TACE and 2 rounds of conventional TACE. In addition, he had a 3.3 cm–sized, partially Lipiodol-laden HCC in S4b of the liver, abutting at the duodenal 1st portion (Fig. 1). Before the procedure, his laboratory results were unremarkable, except for increases in the following biomarkers: lactate dehydrogenase (523 [218–472] U/L), γ-glutamyl transpeptidase (282 [5–61] U/L), C-reactive protein (CRP; 7.55 [0–0.3] mg/dL), α-fetoprotein (8.94 [0.89–8.78] ng/mL), and prothrombin induced by vitamin K absence or antagonist-II (42 [0–40] mAU/mL). His total Child–Pugh score was considered as class A. However, he looked healthy and had no documented fever. He underwent conventional TACE, with the identification of tumor feeders after cone beam CT angiography. Superselective catheterization was applied to the small tumor feeders from the supraduodenal artery and right gastroepiploic artery. Infusion was performed with 10 mg of doxorubicin (Adriamycin) and 2 mL of a Lipiodol mixture followed by embolization with 150- to 350-µm–sized gelatin sponge particles (Fig. 2). He was discharged without acute complications.

**Figure 3.**
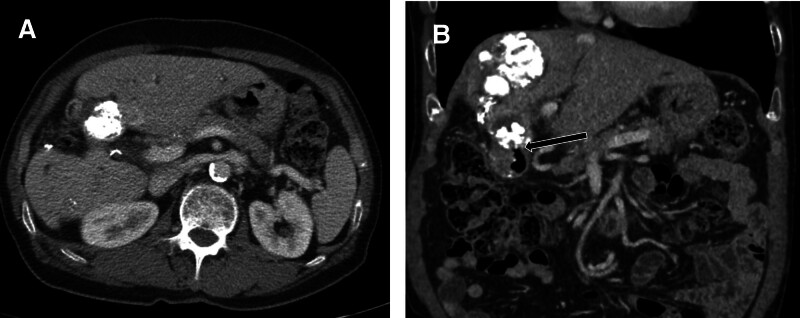
Postoperative CT scans. (A and B) Three weeks after procedure, there was a compact lipiodolized tumor in S4b. A slight protrusion of iodized oil into the duodenal lumen was noted (arrow). CT = computed tomography.

**Figure 4.**
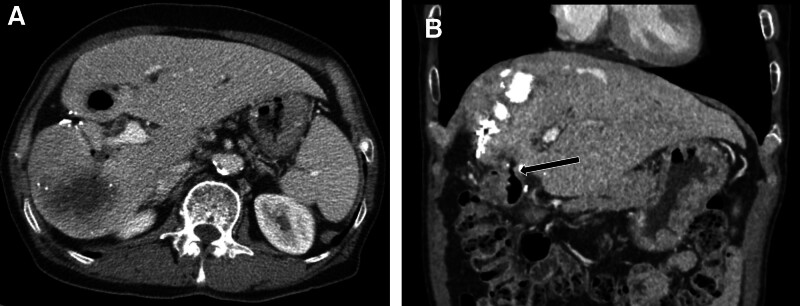
Two-month postoperative CT scans. (A) A 2 cm–sized oval air density lesion instead of a lipiodolized tumor in S4b. (B) Communication between the lesion and the duodenal lumen (arrow). CT = computed tomography.

**Figure 5.**
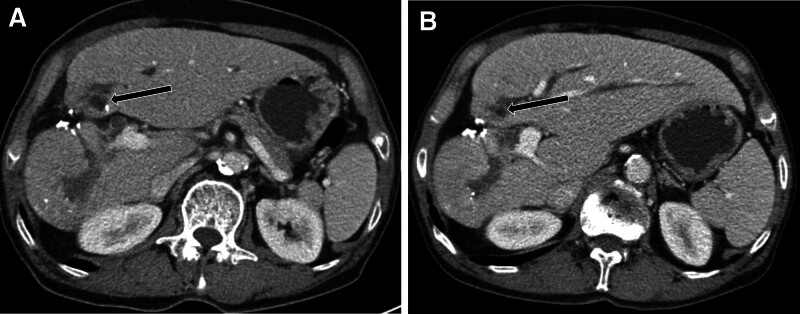
(A) A 1.5 cm–sized low-density lesion in S4b of the liver on 5-mo postoperative CT scan (arrow). (B) Gradual decrease in the size of the lesion over time (arrow). CT = computed tomography.

After 3 weeks, the levels of α-fetoprotein and prothrombin induced by vitamin K absence or antagonist-II were normalized. A follow-up CT scan showed no tumor recurrence; however, compact Lipiodol uptake was observed in the 1st portion of the duodenal wall adjacent to the treated HCC (Fig. 3). Two months later, another follow-up CT scan revealed that the previous Lipiodol-laden HCC had disappeared and a 2-cm air cavity had formed in S4b, which was connected with the duodenal 1st portion (Fig. 4). Subsequent CT scans demonstrated a gradual decrease in the size of the air cavity, with a tiny hypodense focus remaining on the follow-up CT scans (Fig. 5).

At the first outpatient visit following the procedure, the patient complained of intermittent mild fever (<38°C). Laboratory findings showed a normal white blood cell count of 8.0 × 10^3^/µL and an elevated CRP level of 10.45 mg/dL. The patient was treated conservatively. Two months post-procedure, the nonspecific mild fever persisted with laboratory findings indicating mild leukocytosis (11.1 × 10^3^/µL) and an elevated CRP level of 10.77 mg/dL. At 3 months after procedure, the fever resolved, and both white blood cell and CRP levels were gradually improved without any specific treatments. The patient did not experience any further episodes of nonspecific fever over the subsequent 12 months.

## 3. Discussion

The development of a hepatoenteric fistula following TACE alone is an exceedingly rare occurrence, with only a few case reports documented.^[3,7]^ In these reports, the patients had undergone multiple sessions of TACE, and they exhibited obvious symptoms that required hospitalization and intensive medical interventions. In contrast, our case report describes a case in which the hepatoduodenal fistula was initially undiagnosed by hepatologists due to the patient’s nonspecific symptoms and ambiguous laboratory findings. Nevertheless, serial CT scans unequivocally demonstrated the presence of a hepatoduodenal fistula. Following TACE, the patient exhibited nonspecific symptoms, and notably, the fistula was reduced over time without any targeted treatment. To the best of our knowledge, this is the first report of a silent hepatoduodenal fistula following TACE.

The unintentional embolization of adjacent vessels during TACE could result in organ ischemia. Specifically, the embolization of vessels supplying the surrounding gastrointestinal tract can lead to mucosal ulceration or perforation, which may ultimately give rise to hepatoenteric fistula development.^[8]^ Leaving a hepatoenteric fistula untreated may precipitate recurrent ascending infections and potentially result in liver abscess formation, thereby compromising liver function.^[9]^ We identified small branches carrying tumor feeders that might have been unintentionally embolized during superselective angiography and TACE. Although these procedures aim to prevent the inadvertent embolization of neighboring vessels, it is usually impossible to avoid situations where embolization occurs in blood vessels other than those supplying the tumor.

Transarterial embolization of the stomach and duodenum is considered safe because of the rich collateral supply in the area. Specifically, the first portion of the duodenum receives its blood supply from both the supraduodenal artery and retroduodenal artery.^[10,11]^ We embolized the 2 small arteries simultaneously, which could induce proximal duodenal ischemia and wall necrosis despite the duodenum’s collateral circulation, potentially resulting in a hepatoduodenal fistula. This could be attributed to underlying medical conditions, such as the patient’s history of diabetes mellitus. Moreover, repetition of the TACE procedure and previous hepatectomy are potential risk factors for complications following TACE. In our case, serial CT scans showed no signs of abscess formation; consequently, the patient did not require further intervention for the fistula. We speculate that the Lipiodol-laden HCC was expelled through the hepatoduodenal fistula and subsequently resolved. Well-differentiated HCC is characterized by having an outer capsule. It is possible that there was a fistula large enough for the Lipiodol-laden HCC, including its capsule, to enter the intestines, potentially leading to encapsulation in that area.

There are several limitations in this case report. First, as an individual case report, it contains limited research data, necessitating further exploration with appropriate data to determine correlations with other clinical variables. Second, no visual observation or biopsy of the lesion via duodenoscopy was performed during the follow-up period, making it impossible to confirm the pathophysiology of this condition. Third, the heterogeneity of treatment options and management across medical centers worldwide can affect the interpretation of reports. However, to the best of our knowledge, this is the first report of an asymptomatic hepatoduodenal fistula, and the findings may provide crucial information for clinical management in the future.

In conclusion, careful consideration is necessary to avoid embolizing nontarget vessels when a target tumor is near other organs. However, even with precise target embolization, close monitoring is required after treatment, as asymptomatic hepatic fistulas may occur despite their rarity.

## Author contributions

**Investigation:** Byung Chan Lee.

**Writing – original draft:** Byung Chan Lee, Chan Park.

**Writing – review & editing:** Byung Chan Lee, Hyoung Ook Kim, Chan Park, Inwoo Choi, Seung Wan Kang.

**Project administration:** Chan Park.

**Supervision:** Chan Park.
